# Hip joint motion does not change one year after arthroscopic osteochondroplasty in patients with femoroacetabular impingement evaluated with dynamic radiostereometry

**DOI:** 10.1186/s40634-021-00427-x

**Published:** 2022-01-05

**Authors:** Lars Hansen, Sepp de Raedt, Peter Bo Jørgensen, Bjarne Mygind-Klavsen, Lone Rømer, Bart Kaptein, Kjeld Søballe, Maiken Stilling

**Affiliations:** 1 Aarhus University Hospital, Aarhus, Denmark; 2 Leiden University Medical Center, Leiden, Netherlands

**Keywords:** Hip, FAI, Femoroacetabular impingement, Hip biomechanics, Arthroscopic osteochondroplasty

## Abstract

**Purpose:**

Dynamic radiostereometric analysis (dRSA) enables precise non-invasive three-dimensional motion-tracking of bones for assessment of joint kinematics. Hereby, the biomechanical effects of arthroscopic osteochondroplasty of the hip (ACH) can be evaluated in patients with femoroacetabular impingement (FAI).

The aim was to investigate the pre- and postoperative range of motion (ROM) and the CT bone volume removed (BV) after ACH. We hypothesize increase in ROM 1 year after surgery.

**Methods:**

Thirteen patients (6 female) with symptomatic FAI were included prospectively. The patient’s hips were CT-scanned and CT-bone models were created. Preoperative dRSA recordings were acquired during passive flexion to 90°, adduction, and internal rotation (FADIR). ACH was performed, CT and dRSA were repeated 3 months and 1 year postoperatively. Hip joint kinematics before, and 3 months and 1 year after ACH were compared pairwise. The bone volume removal was quantified and compared to change in ROM.

**Results:**

Mean hip internal rotation, adduction and flexion were all unchanged after ACH at 1-year follow-up (*p* > 0.84). HAGOS scores revealed improvement of quality of life (QOL) from 32 to 60 (*p* = 0.02). The BV was between 406 and 1783 mm^3^ and did not correlate to post-operative ROM.

**Conclusions:**

ACH surgery in FAI patients had no impact of ROM at 1-year follow-up. QOL improved significantly. This indicates that the positive clinical effects reported after ACH might be a result of reduced labral stress and cartilage pressure during end-range motion rather than increased ROM.

**Level of evidence:**

Therapeutic prospective cohort study, level II.

## Introduction

Femoroacetabular impingement (FAI) is caused by excess bone on the acetabular rim (pincer- type), by excess bone on the proximal femur (cam-type) or by a combination of the two (mixed-type) [1, 2]. FAI most often presents in healthy, physically active, young persons (predominantly male) in the age range of 20–30 years [14]. It is a precursor for early development of osteoarthritis and a common cause of pain [24, 26]. The reported prevalence of asymptomatic FAI in radiographs is 23–32% for CAM lesions and 43–67% for pincer lesions [10, 12]. Studies show that physical impairments for individuals with symptomatic FAI primarily consist of motions bringing the hip towards impingement [7–9, 13].

The surgical treatment of FAI is arthroscopic osteochondroplasty on the femoral and/or acetabular side (ACH) [29]. Excess bone is removed in the corresponding areas of impingement at the femoral neck and acetabular rim. The primary cause for reoperation after ACH procedure is failure to identify and/or adequately reshape the affected bone areas within the joint [30].

Clinical studies of hip joint kinematics in FAI patients are limited. Some studies using CT-bone models for simulation of impingement positions exist, but the clinical value is limited as they do not describe the in vivo joint motion or correlations with clinical symptoms [3, 25, 35]. Studies using motion capture systems primarily investigate in-vivo hip kinematics during gait or squat [5, 9, 33, 36]. Kapron et al. [20, 21] showed that FAI-patients have decreased adduction and internal rotation during passive tests. Also, they showed that ROM is governed by soft tissue restraints and not by bone-bone contact, but postoperative kinematics were not investigated.

The in vivo pathomechanics of FAI are not well understood, and neither are the effects of ACH surgery on hip kinematic. There has been a request for methods to evaluate the objective kinematic changes in the hip joint following ACH in order to provide evidence of the efficacy of surgery [3]. We have previously validated dynamic radiostereometric analysis (dRSA) for precise objective assessments of hip kinematics by use of CT bone volume models [15, 16].

The aim of this clinical study was to compare hip-joint kinematics before and after ACH in FAI patients by use of dRSA in order to understand the beneficial effects of FAI surgery. We hypothesize increase in ROM 1 year after surgery.

## Methods

### Design

The present study is a prospective longitudinal cohort study comparing range of motion, radiological measures, and the bone volume of the hip before and after ACH. This was done using CT bone-models in combination with dRSA for thorough investigation of in vivo hip joint kinematics. The study was approved by The Central Denmark Region Committees on Health Research Ethics (Case number M-2015-270-15) and performed in accordance with the Helsinki II declaration.

### Participants

Thirteen patients (6 female) diagnosed with uni- or bilateral FAI were included. Inclusion criteria were a diagnosis of CAM- and/or pincer impingement (alpha angle > 55 degrees and/or CE-angle > 40 degrees), planned hip arthroscopy. Exclusion criteria were hip dysplasia (CE < 25 degrees and acetabular index angle ≥10), previous hip arthroscopy or arthroplasty, neurological diseases, cancer, pregnancy at inclusion, and reoperation.

### Physical examination

All patients underwent a thorough physical examination by the surgeon prior to ACH, including prior medical history, medication, hip examination, and an overall evaluation of general health. The FADIR test was considered positive if pain was elicited in full flexion, adduction and internal rotation as described by Byrd [6]. Patient reported outcome measures (PROMs) were collected using the Copenhagen Hip and Groin Outcome Score (HAGOS) [37].

### Surgical technique

A traditional intraoperative hip arthroscopy setup was used with patients in a supine position on a traction table. ACH was performed with a 70° wide angle arthroscope, a radiofrequency wand (Super Multivac 50, Smith and Nephew, London, United Kingdom), burr (5.5 mm barrel burr) and a shaver (Dyonics Incisor Plus, Smith and Nephew, London, United Kingdom). The osteochondroplasty was performed by an experienced arthroscopist. During surgery the surgeon restored what appeared to be clinically normal morphology (guided by intraoperative flouroscopy) in accordance with the Danish Hip Arthroscopy Registry (DHAR) (average circumferential area of 116° (SD = 24.5) and a mean depth of 3.8 mm (SD = 1.7) [27].

### Imaging

#### Ct

Preoperative CT scans (Brilliance 64, Philips Medical Systems, Best, The Netherlands) of the pelvis and proximal femur were acquired. Settings were 120 kV, 150 mAs, slice thickness 2.5 mm and slice increment 1.25 mm. Bone volume models were produced and all segmentations were verified to be within voxel accuracy (< 0.3 mm) [16]. Local coordinate systems were created for the bone models by the method described by Wu et al. [38]. Postoperative CT-scans included the acetabulum and proximal femur. All CT scans were clinically evaluated by one senior radiologist and relevant radiological measures were obtained in 1 plain: center edge angle (CE), alpha angle, acetabular index, femoral anteversion.

#### Radiographic setup

**Fig. 1:**
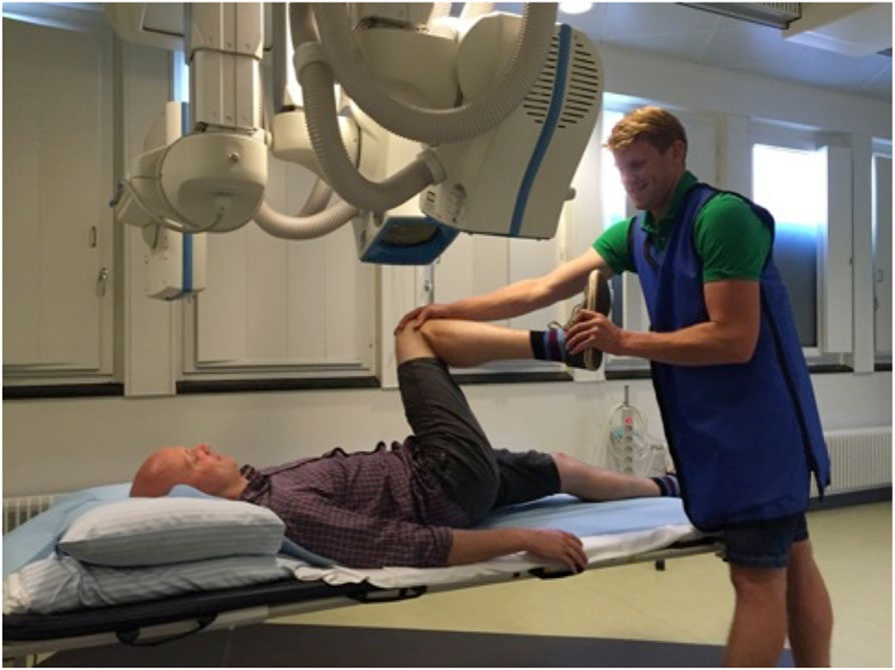
Clinical examination of patient during the FADIR test. The image also showing the radiographic setup

All stereoradiographs were recorded using a dynamic RSA system (Adora RSAd, NRT X-Ray, Denmark). Sampling frequency was five frames/s for approximately 8 s, pulse width 16 ms. Roentgen tubes were positioned with a 45° cranio-caudal- and 20° medio-lateral tilt directed at the hip joint from the cranial-caudal direction. A uniplanar calibration box (Box 14; Medis Specials, Leiden, the Netherlands) was placed in a 45° angle to the horizontal plane (Fig. 1). The two image detectors (Canon CXDI-50RF) were slotted in the calibration box. Source image distance (SID) was 2220 mm and focus skin distance (FSD) 1140 mm. Exposure settings for dRSA recordings were 130 kV, 500 mA, 16 ms and resolution was 1104 × 1344 pixels (79 dots per inch) [15].

#### Dynamic RSA

dRSA recordings were acquired during passive hip motion by the examiner with flexion to 90°, adduction to stop and internal rotation to stop (FADIR) as validated by Hansen et al. [15] (Fig. 1). During the 8 s recording the FADIR motion was repeated approximately three times. Postoperative dRSA recordings were acquired at 3- and 12 months follow-up. Calibration of the sequences was performed by calibrating the first image in each sequence using model-based RSA (RSAcore, Leiden, Netherlands).

#### Digitally reconstructed radiographs

**Fig. 2:**
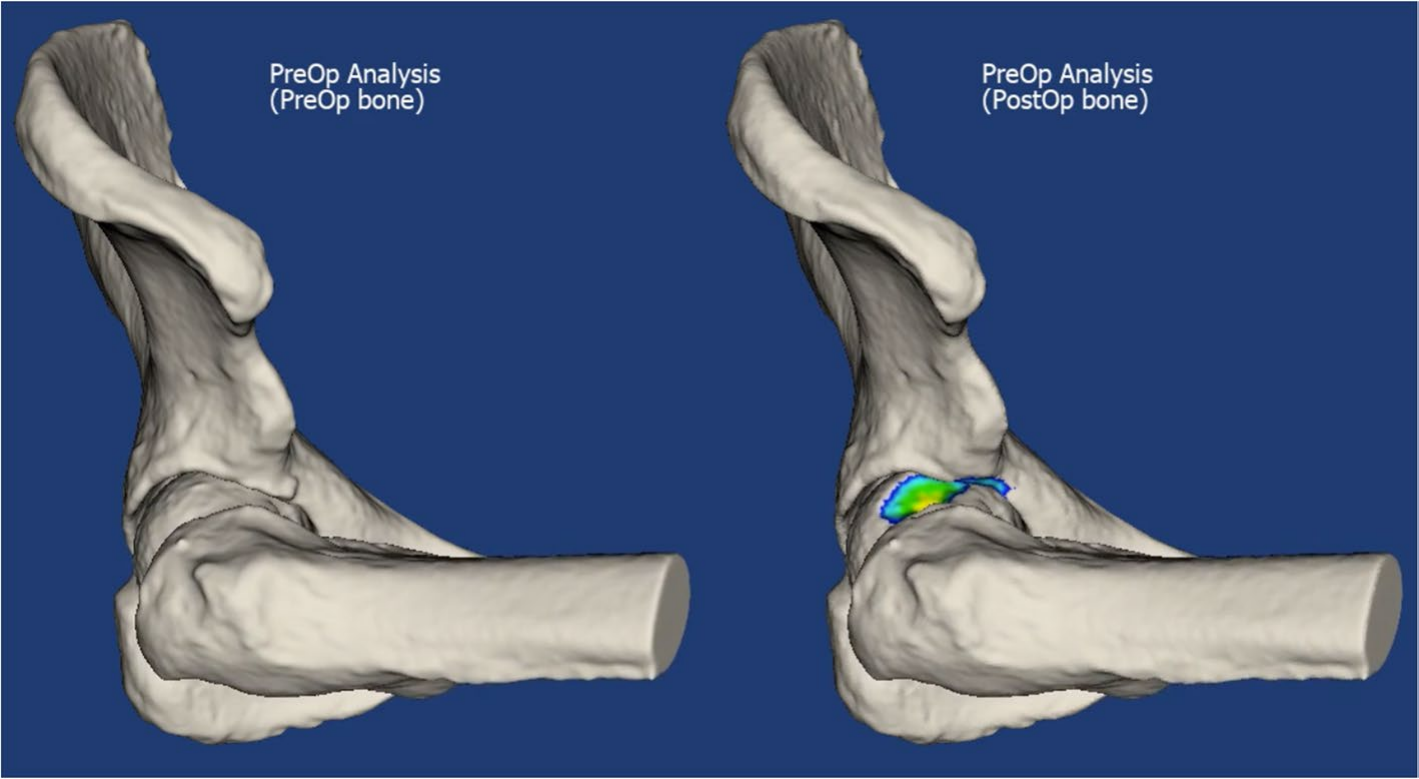
Fitting of the bone using the DRR technique. Colored area marks the area of removed bone

All dRSA recordings were analysed using a customized automatic analysis method based on digitally reconstructed radiographs as described by Hansen et al. [15]. The automated DRR analysis is based on intensity-based two-dimensional and three-dimensional image registration using the volume model. The 3D CT bone volume model was used to simulate images, which were then compared to the RSA images using a similarity metric. The position and orientation with best similarity metric, corresponds to the optimum position (Fig. 2).

#### Precision of dRSA using DRR

In an experimental study on human hip specimens Hansen et al. showed that the DRR method had a precision below 0.32 mm for translation and 0.36° for rotations for analysis of dRSA images of hip kinematics [16]. This precision is sufficient for revealing clinically relevant information of hip joint kinematics.

#### Bone volume

The volume of removed bone during ACH was determined by aligning the pre- and post-operative CT scans using image registration and segmenting the region with an intensity difference above 50 Hounsfield units [23]. Resulting in a bone model representing removed bone and quantified in mm^3^ for each patient.

#### Radiation dose

Based on real time dRSA recordings dose-calculations were performed. The revealed effective dose per exposure was 0.054 mSv. Recordings were acquired at 5 frames/s with a mean exposure time of 8 s, resulting in an effective dose of 2.43 mSv per recording. The CT-scan contributed with an effective dose of 5.2 mSv for the preoperative scan and approximately 3 mSv for the postoperative scan due to a reduction of the field of view. The total effective dose for all follow-ups in the study was 15.5 mSv per patient.

#### Data analysis and statistics

The frames (dRSA images) with the hip in end-range FADIR position (mean of 3 per recording) were identified and defined as the highest rotation for each FADIR motion cycle. Data normality was assessed on QQ plots. Hip joint kinematics before and after ACH were compared pairwise with one-way ANOVA testing using the Bonferroni correction. The volume of removed bone was quantified and correlated to change in hip range of motion (ROM) by use of Spearman’s rho. Radiological angles and translations of the bone in end-range position were compared using a paired t-test. Subluxation of the femoral head center in the acetabulum was measured as translations along the x-, y- and z-axis and calculated as a combined measure of total translation = ($\sqrt{x^2+{y}^2+{z}^2}$). The preoperative and 1-year HAGOS results were compared using the paired t-test. Statistical analyses were performed using Stata/IC 14.1 (StataCorp, College Station, Texas, USA).

We did not perform a power calculation prior to initiation. To our knowledge no other studies have used RSA to evaluate the hip joint pre- and postoperatively. The technique used in this study is very accurate and the low number of patients included should be sufficient to show a clinical relevant difference between pre- and postoperative [15, 16].

#### Patient and public involvement

There was no patient/public involvement in designing the study.

## Results

**Fig. 3:**
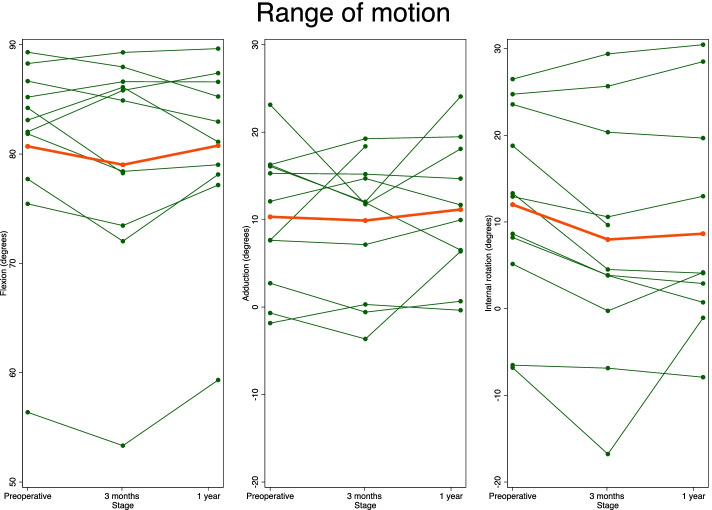
Development in flexion-, adduction- and internal from preoperative to 3-months and 1-year follow-up. Red plot showing the mean development for all patients

Thirteen patients (6 female) diagnosed with uni- or bilateral FAI were included. Two patients were excluded due to excessive weight and poor image quality and one missed the 1 year follow-up due to pregnancy. Patient demographics and results are listed in Table 1. No statistically significant or clinically relevant difference in end-range passive FADIR ROM was observed from before to after surgery. Internal hip rotation was mean (CI95%) 11.4° (3.58–19.3) before surgery, 7.83° (− 1.44–17.1) at 3 months, and 8.9° (0.074–17.7) at 1 year after ACH surgery, (*p* > 0.84) (Fig. 3). Hip adduction was mean (CI95%) 9.2° (3.54–14.9) preoperatively, 9.6° (4.29–14.8) at 3 months, and 10.5° (5.06–15.9) at 1 year after ACH surgery (*p* > 0.87) (Fig. 3). Hip flexion was used as a measure of reproducibility of the first step in the passive FADIR motion during dRSA examinations. Hip flexion was mean (CI95%) 80.9° (74.8–87.1) preoperatively, 79.4° (72.5–86.4) at 3 months, and 80.3° (74.6–86.0) at 1 years after ACH surgery (*p* > 0.94) (Fig. 3). Subluxation of the femoral head center in the acetabulum in terms of total translation was 1.28 mm (CI95%. 0.82–1.75) before surgery and similar after ACH surgery (*p* > 0.8).

**Table 1:** Patient demographics

Patient number	Age	Gender	BMI	Removed bone pelvis (mm^3^)	Removed bone femur (mm^3^)	Flexion (°)	Adduction (°)	Internal rotation (°)	Caput ICRS	Acetabulum Becks
1	46	Female	23.6	216	984	1.39	7.98	−7.91	1	3
3	39	Female	22.6	313	478	−2.83	−9.74	−1.39	1	3
4	21	Male	24.8	166	883	−4.05	−5.04	5.75	1	5
5	53	Male	21.8	220	1563	5.37	2.32	−5.31	1	4
6	39	Male	25.5	0	1576	2.31	−0.41	−3.89	1	4
7	27	Female	19.4	244	1028	–	–	–	1	3
8	51	Female	20.3	102	690	−3.70	3.19	−0.97	4	4
9	41	Male	22.3	341	406	0.42	−2.06	3.97	2	2
10	26	Female	19.8			1.72	−0.61	3.75	1	2
11	58	Female	24.6	411	428	−1.99	7.05	0.04	2	3
12	26	Male	23.5	199	319	1.36	1.48	−9.21	1	4

Flexion: change in flexion from preoperative to 1 year postoperative

Adduction: change in adduction from preoperative to 1 year postoperative

Internal rotation: change in internal rotation from preoperative to 1 year postoperative

Caput ICRS: The International Cartilage Repair Society Grade of the femoral head [11]

Acetabulum Becks: Beck Classification of Cartilage damage of the acetabulum [28]

*BMI* Body mass index

- : Patient 7 was lost to one year follow-up due to pregnancy

**Fig. 4:**
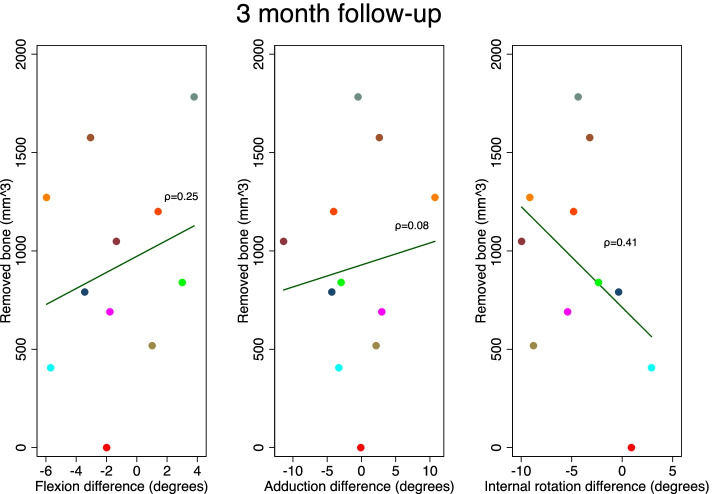
Flexion-, adduction- and internal rotation difference is the difference between the achieved angle preoperatively and at 3 months. Spearman’s rho values in parenthesis. No significant correlation was found. Colors of plots specific for each patient

**Fig. 5:**
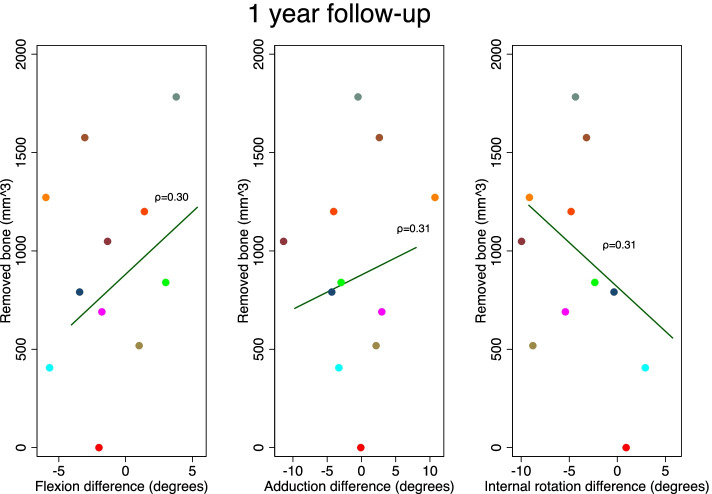
Flexion-, adduction- and internal rotation difference is the difference between the measured end-range angle preoperatively and at 1 year. Spearman’s rho values in parenthesis. No significant correlation was found. Colors of plots specific for each patient

The resected BV was mean 909 mm3 (SE 90 mm3) with a range between 406 and 1783 mm3. The Spearman’s rho revealed no correlation between BV and range of motion (Spearman’s rho 0.025; *p* = 0.91) (Figs. 4 and 5). The radiological measures were similar before and after ACH surgery (Table 2).

**Table 2:** Radiological measures before and after ACH surgery measured on CT radiographs

Measure	Preoperative (SD)	Postoperative (SD)	***P***-value	Limits
**CE**	31.8 (1.9)	31.6 (1.9)	0.94	25–39°
**Alpha**	61.3 (8.8)	51.6 (4.1)	0.33	> 55°
**AI**	2.7 (1.6)	3.3 (2.2)	0.83	0–10°
**FeAv**	24.2 (9.0)	21.9 (8.2)	0.58	5–20°

Limits: Normal ranges [4, 10, 34]

*CE* Center edge angle, *Alpha* Alpha angle, *AI* Acetabular index, *FeAV* femoral anteversion

Ten patients responded to preoperative HAGOS and 7 to the 1-year postoperative. The PROMs revealed an increase on all parameters 1 year after ACH surgery with significant improvement in quality of life from 32 (3.1) to 60 (12) (*p* = 0.02) (Table 3).

**Table 3:** HAGOS scores before and 1 year after ACH surgery

	Pain	Symptoms	ADL	Sport & recreation	PA	QOL
**Preoperative** (*n* = 10) mean, SD	61 (4.8)	51 (3.7)	55 (6.7)	39 (9.2)	24 (9.8)	32 (3.1)
**Postoperative** (*n* = 7), mean, SD	75 (9.8)	63 (7.5)	75 (11)	62 (10)	52 (14)	60^a^ (12)

*ADL* Activities of daily life, *PA* Physical activity, *QOL* Quality of life

^a^: Statistically significant

## Discussion

We found similar hip ROM in flexion, adduction and end-range internal rotation between preoperative and 1 year after ACH surgery. Preoperative subluxation was measured and did not change after surgery indicating preserved hip joint stability. Hip flexion angles were reproducible in all patients with a little variation between patients. Small variations might be explained by pelvic tilt since hip flexion was anticipated to be 90° when the thigh was orthogonal to the underlying surface. Radiological measures were unchanged including the CE and the alpha angle. The alpha angle was lower than the radiological limit, though not significantly. PROMs reported using HAGOS scores improved on all parameters with significant improvement of quality of life. The HAGOS scores correlate well with previous observations in the HAFAI cohort [18, 22, 31].

Subluxation and ROM did not change clinically relevant or statistically significant after surgery and the reason for the positive effects of ACH remains to be found. Johnston et al. have shown that there is a relationship between offset of the alpha angle and the prevalence of labral and chondral damage [19]. A possible explanation could be that resection of cam and pincer deformities reduces stress on soft tissue structures such as the labrum or cartilage, and just enough to prevent repeated damage but without influencing passive ROM. Although no difference in subluxation was observed it cannot be ruled out that there could be decreased lever function of the femur in some functional positions of the joint. Subsequently, these areas of impingement would have been missed in the current study.

Kapron et al. have performed a study, which was comparable to ours in examined hip motion during passive examiner induced FADIR, and recording/analyzing with a precise DRR dRSA method [20]. The study only included a kinematic evaluation of six normal controls and three FAI patients preoperatively. No statistical comparison was presented due to the low number of subjects. For the three symptomatic FAI patients, the internal rotation during FADIR was between mean 7.8° at preoperative and at 11.6° 1 year postoperatively, which is comparable to the preoperative internal rotation of 11.4° found in this study. Adduction during the FADIR test was 2.2° – 5.7° which is lower compared to the findings of this current study. Due to low numbers and high variance these values are difficult to compare directly.

Rylander et al. and Hunt et al. have studied the functional kinematics of FAI in a gait lab and have shown that patients with symptomatic FAI have reduced ROM during gait and stairclimbing [17, 32]. In another gait lab study Brisson et al. have investigated the functional kinematics of FAI pre- and postoperatively without finding improvements, whereas Rylander et al. have shown normalizing gait patterns at 1 year postoperative [5, 33].

The dRSA method used is extremely precise for investigation of in-vivo hip function in a clinical study. To our knowledge the current study is the only study investigating pre- and postoperative FAI hip function with a high precision method. The setup was designed to analyze hip function during the FADIR impingement test.

A significant limitation related to the study is the high radiation dose. Although major dose reductions have been achieved during the course of the study the exposure per recording is still 2.43 mSv and to this must currently be added the dose of the CT scan. With our increasing experience in dRSA of the hip it was possible to reduce the preoperative CT scan-field significantly. Further, we were able to reduce the postoperative scan-field to only include the area in which bone was removed to determine BV and use the preoperative scan for the postoperative dRSA kinematic analyses. Consequently, it would be possible to completely leave out the postoperative CT in case the clinician only is interested in pre- and postoperative ROM and subluxation – and not in resected bone volume. In a future perspective, bone models from low-dose imaging systems such as cone beam CT and MRI may further reduce radiation exposure. The FADIR test is influenced by the clinician and therefor results might differ. We attempted to minimize the examiner effect of FADIR by performing the FADIR test three times at each recording and use the mean of three image frames. The limited number of patients included is another limitation of this study.

Radiological angles were measured in one plane on CT, why they might be significantly reduced in areas where we have not measured. ROM was possibly correlated to BV at 1-year follow-up, although not conclusive as there are too few measurements to perform a regression analysis.

## Conclusions

In conclusion, dRSA was a useful and precise clinical method to evaluate hip joint kinematics and may contribute with valuable information of pre- and postoperative hip joint kinematics. We found no measurable improvements of FAI surgery on hip kinematics. This indicates that the pain reduction of FAI surgery is not brought by increased ROM. Possible factors could be labrum stabilization or reduced stress on the labrum and/or cartilage. These findings can be useful in the preoperative information to patients, which makes this study important.

## Abbreviations

ACH: Arthroscopic osteochondroplasty
BMI: Body mass index
BV: Bone removed
CE: Center edge angle
DHAR: Danish Hip Arthroscopy Registry
DRR: Digitally reconstructed radiographs
dRSA: Dynamic radiostereometric analysis
FADIR: Flexion, adduction and internal rotation
FAI: Femoroacetabular impingement
PROM: Patient reported outcome measure
QOL: Quality of life
ROM: Range of motion
RSA: Radiostereometric analysis
SD: Standard deviation
SE: Standard error
